# A dataset on the features of the elimination of explosive objects using a dome-shaped protective device with a load

**DOI:** 10.1016/j.dib.2023.109602

**Published:** 2023-09-21

**Authors:** Denis Lyovin, Victor Strelets, Roman Shevchenko, Valentyna Loboichenko, Mikhail Divizinyuk, Valery Strelets, Andrei Pruskyi

**Affiliations:** a National University of Civil Defence of Ukraine, Chernyshevska Str., 94, Kharkiv 61023 Ukraine; b Universidad de Sevilla, Calle San Fernando, 4, Seville 41004, Spain; c Lutsk National Technical University, Lvivska Str., 75, Lutsk 43018, Ukraine; d Institute of Environmental Geochemistry of the NAS of Ukraine, Academician Palladin Ave. 34-А, Kyiv 03142, Ukraine; e International Humanitarian Organization The Halo Trust in Ukraine, Reytarska Str., 21/13, r. 23, Kyiv 01034, Ukraine; f Institute of Public Administration and Civil Protection Research, Rybolovnaya Srt., 18, Kyiv 01011, Ukraine

**Keywords:** Explosive object, Protective device, Liquidation, Emergency prevention

## Abstract

In this article, the results of experimental explosive research of a dome-shaped protective device (made of St20 steel, 90 cm diameter, 130 kg weight) with a load have been given. These studiesused standard statistical procedures with a 0.95 reliability level to establish the validity of the existing mathematical model for emergency prevention. These are associated with the threat of the explosion of a small-sized explosive object. That allowed to substantiate the features that must be taken into account by the personnel of pyrotechnic divisions in operational activities (compliance with the additional load on the body of small-sized explosives trinitrotoluene (TNT), the need for passive protective embankment, stock in the pyrotechnic division of protective load, for example in the form of bags with sand).

## Graphical abstract

**Figure:**
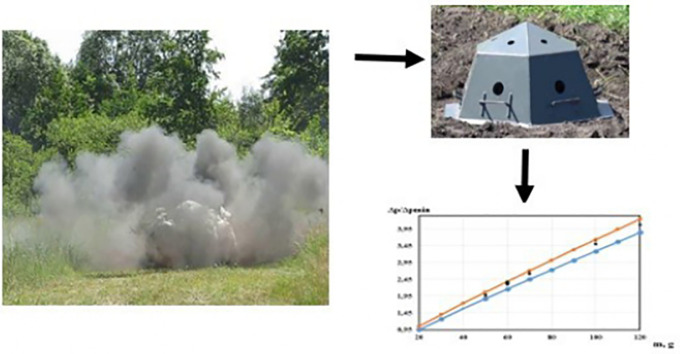

Specifications Table

**Table:** 

Subject	Engineering
Specific subject area	Military Engineering
Data format	Raw, Analyzed. .csv files (datasets with numbers) .docx file (datasets with numbers)
Type of data	Table, Image, Graph, Figure, Text document
Data collection	The data were acquired through experimental explosive studies of a dome-shaped protective device (made of St20 steel, diameter 90 cm, weight 130 kg) [9] with an additional load as a result of the explosion of a small-sized explosive object. The overpressure (Table 1) that occurs inside the means of localization at different values of the mass of the charge (namely in the range from 30 to 120 g of TNT) is calculated. In order to determine overpressure during full-scale explosive tests, additional equipment was mounted on the localization device (Fig. 2), which allowed for the obtaining indicators of overpressure that occurred inside the protective device and recording them in a timely manner. A manometer of the MT-УХЛЗ type was used.
Data source location	Institution: National University of Civil Defence of Ukraine City/Town/Region: Kharkiv region Country: Ukraine
Data accessibility	Repository name: Mendeley Data Data identification number: 10.17632/2yxprr6sz2.2 Direct URL to data: https://data.mendeley.com/datasets/2yxprr6sz2/2

## 1 Value of the Data

Today, the issue of liquidation of small-sized explosive objects is acute, where liquidation can only be carried out at the place of detection.

The presented dataset makes it possible to study the effect of the protective device in the process of eliminating small-sized explosive objects, and, due to the paucity of such information, are of increased value.

This dataset is of value to a wide range of specialists involved in the disposal of small-sized explosive objects at the site of detection, in particular, pyrotechnicians of special services, humanitarian demining specialists. The article verifies the reliability of a mathematical model for preventing emergencies associated with the threat of an explosion of a small explosive object, which is based on the use of a protective device with an additional load on the body, by conducting a real fire explosive test.

This dataset can be used in further research related to the elimination of explosive objects of various masses to minimize the consequences of hostilities and possible terrorist acts, including the development of new methods and means of eliminating explosive objects and the development of mathematical models for the activities of specialists in their elimination.

## 2 Objective

Hostilities, military conflicts, terrorist acts have a significant negative impact on the population, infrastructure, and environment [1,2]. Currently, in the field of explosion protection technology, a wide range of devices is used for transporting, neutralizing, and destroying explosive objects [3]. They are designed to localize explosive products of high-explosive fragmentation in the event of a possible detonation of an explosive device of a certain power and mass [4,5] and can be described by reliable mathematical models [6], [7], [8]. However, the use of explosion-proof equipment without additional load poses a danger to the environment and the personnel of the pyrotechnic unit.

The purpose of the work is to verify the reliability of the mathematical model for preventing emergencies associated with the threat of an explosion of a small-sized explosive object. The model is based on the use of a protective device with an additional load on the hull after conducting real blast tests on the prototype. As a result, features will be identified that, together, will ensure the development of an appropriate operational and technical methodology for the personnel of pyrotechnic units, further improve approaches to ensuring the safety of specialists involved in demining, etc.

## 3 Data Description

**Fig. 1:**
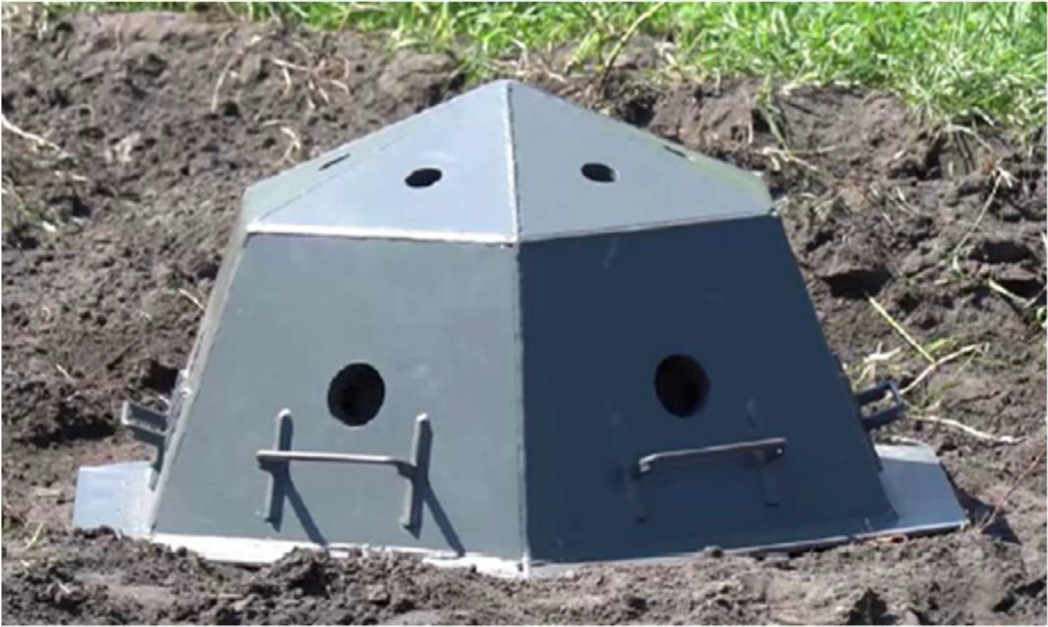
The dome protection device.

To obtain experimental data, the device shown in Fig. 1 was used [9].

**Fig. 2:**
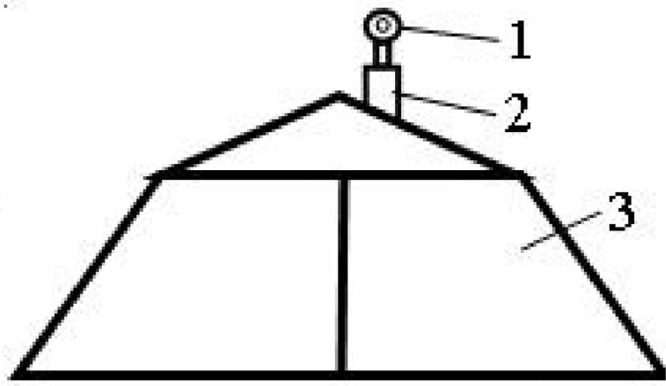
Scheme of acquisition experimental results (1 - pressure gauge; 2 - check valve; 3 – protection device).

Additional equipment was installed to determine the overpressure during field explosive tests on the localization device in accordance with Fig. 2.

Using the developed mathematical model [8] the calculation of overpressure (Table 1, [15]) inside the localization device for different values of the charge mass within its change as a small-sized explosive object (from 30 g to 120 g of TNT) was performed.

**Table 1:** Calculation overpressure results inside the protective device.

m, g	20	30	40	50	60	70	80	90	100	110	120
$\Delta p_{calcul},$ MPа	1,57	2,09	2,60	3,09	3,57	4,05	4,52	4,99	5,44	5,90	6,36

**Fig. 3:**
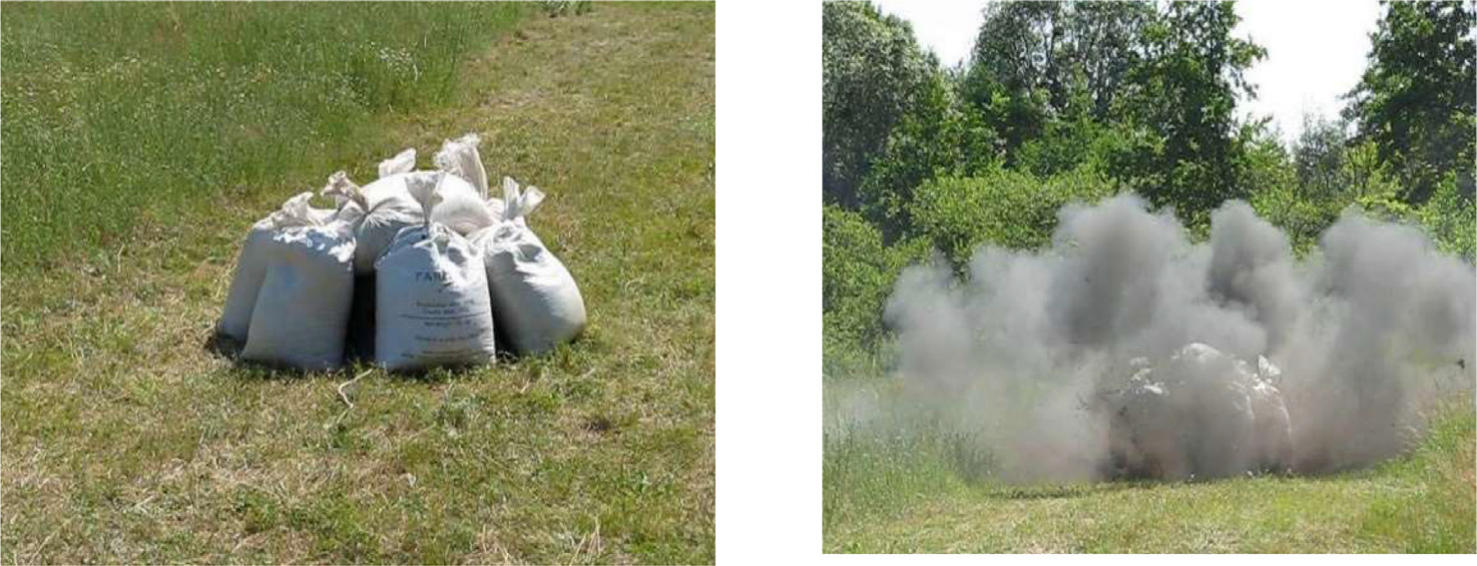
Test of protective device with load.

In the course of carrying out experimental studies on overpressure inside the protective device with an additional load, 15 explosive tests were carried out according to the scheme shown in Fig. 3 and Table 2, [15].

**Тable 2:** Scheme of explosions inside a protective device.

proof №	1	2	3	4	5	6	7	8	9	10	11	12	13	14	15
Mass of TNT, g	50	50	100	60	60	60	60	60	60	60	100	70	70	100	120
The calculating mass of the additional load, kg	214	214	742	314	314	314	314	314	314	314	742	426	426	742	847
Actual mass of the additional load, kg	250	250	750	350	350	350	350	350	350	350	750	450	450	750	850

**Fig. 4:**
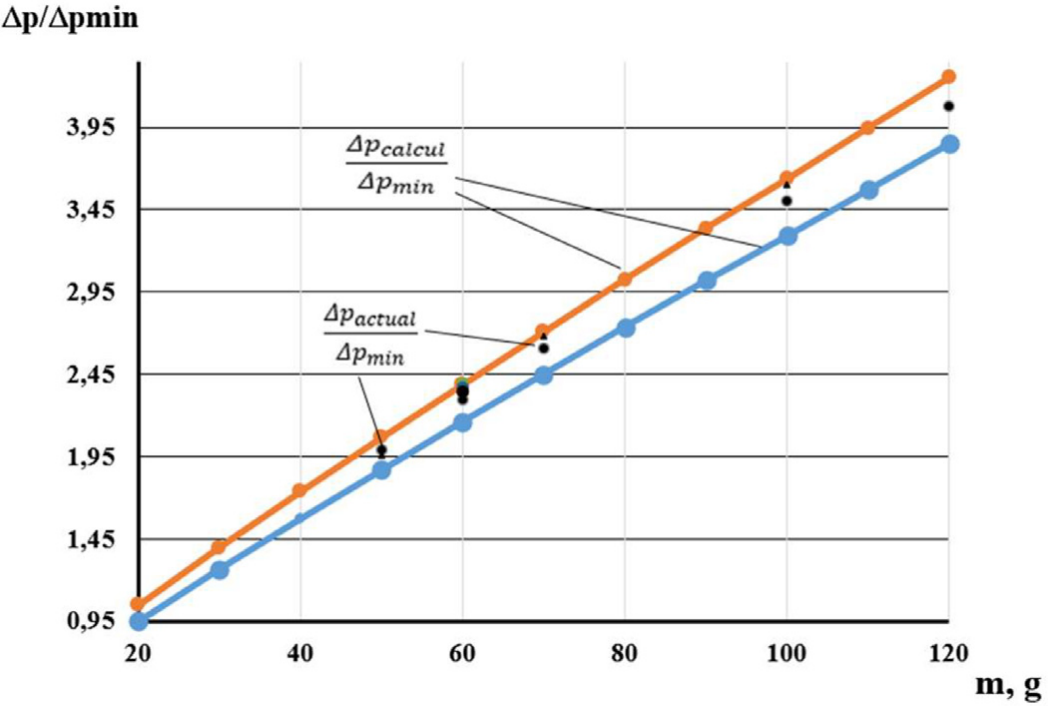
Verification of the conclusion experimental results in the interval ± 5 % in relation to the calculated indicators.

The experimental results obtained were entered in Table 3, [15]. It is more obvious to compare the obtained experimental results to the calculated value of overpressure inside the protective device (Table 4, [15]). This corresponds to 20g of the TNT mass charge. The analysis of hits in the interval of ± 5 % relative to similar calculated results is shown in Fig. 4 and in Table 5, [15].

**Table 3:** Experimental results of determining the overpressure inside the protective device under load.

proof №	1	2	3	4	5	6	7	8	9	10	11	12	13	14	15
m, g	50	50	100	60	60	60	60	60	60	60	100	70	70	100	120
$\Delta P_{calcul},\text{MPa}$	3,09	3,09	5,44	3,57	3,57	3,57	3,57	3,57	3,57	3,57	5,44	4,05	4,05	5,44	6,36
$\Delta P_{\text{actual}},\text{MPa}$	3,13	3,07	5,5	3,61	3,54	3,53	3,61	3,55	3,52	3,52	5.4	4,1	4,02	5,52	6,4
X	1,01	0,99	1,01	1,01	0,99	0,99	1,01	1,00	0,99	0,99	0,99	1,01	0,99	1,01	1,01
**Generalized estimates in coded form**															
$\bar{x}$	1,000														
$\sigma_{x}$	0,010														
$x_{\min}$	1,01														
$x_{\max}$	0,99

**Table 4:** The values of the overpressure inside the protective device, obtained from experimental results.

proof №	1	2	3	4	5	6	7	8	9	10	11	12	13	14	15
$\frac{\Delta p_{actual}}{\Delta p_{min}}$	1,99	1,96	3,50	2,30	2,36	2,35	2,39	2,37	2,35	2,35	3,60	2,61	2,68	3,64	4,08

**Fig. 5:**
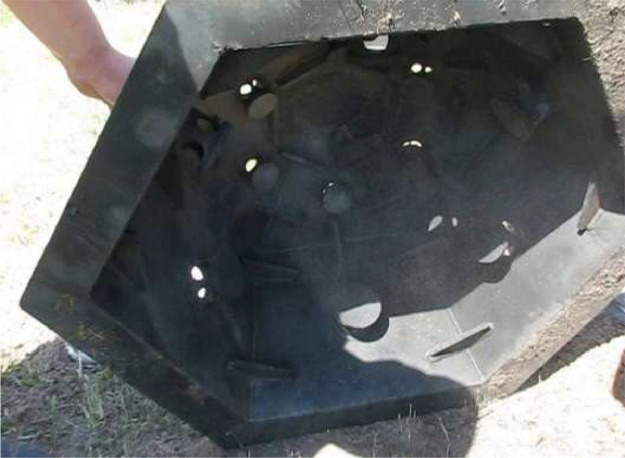
Protective device inside after explosion.

The external inspection of the protective device after each explosion was carried out (Fig. 5).

**Table 5:** Calculated values of the reduced indicator of overpressure inside the protective device and the limits of the ±5 % interval.

m, g	20	30	40	50	60	70	80	90	100	110	120
$\frac{\Delta p_{calcul}}{\Delta p_{min}}$	1	1,34	1,66	1,97	2,27	2,58	2,88	3,18	3,47	3,77	4,06
α=−0,05	0,95	1,26	1,57	1,87	2,16	2,45	2,74	3,02	3,29	3,57	3,85
α=+0,05	1,05	1,40	1,74	2,07	2,39	2,71	3,02	3,34	3,64	3,95	4,25

## 3 Experimental Design, Materials and Methods

### 3.1 Real Protective Device

The study on a real protective device has been carried out. It was made of St 20 steel. The device has 12 faces with holes with a diameter of 38–64 mm (Fig. 1 and 2) on each face. From the inside, the holes are protected by metal flanges of the same material (Fig. 5) to prevent possible fragments of an explosive object from flying out (Fig. 3). When a load (sandbags) was applied, each hole was not tightly blocked. A detailed scheme of the device is presented in the patent [9].

An MT-УХЛЗ (0...250) manometer (Open Joint Stock Company “Teplokontrol”) was used in the research (range 0-25 MPa, division value 0.1 MPa). The pressure gauge fitting is located in the upper part of the device, in one of the holes (Fig. 2). To fix the overpressure with a manometer, a check valve is used (Fig. 2,2). The initial pressure on the pressure gauge is 0.1 MPa (factory specification).

In the experiments, trotyl monoliths (trinitrotoluene TNT) of a rectangular shape and a given mass were used, selected from the original T-400 pressed TNT block (Pavlograd Chemical Plant) and provided by pyrotechnic specialists.

As a detonator, an industrial electric detonator "EDP" (Shostka state-owned plant "Impulse") was used, which was placed inside the explosive samples.

When determining the tactical and technical requirements for the device, the following were taken into account:

- a small-sized explosive object is an explosive object with a TNT 120 g weight of up, which corresponds to most possible situations [10] in which it is necessary to localize or destroy an RG-6 hand grenade on the spot; its TNT weight is 60 g;
- the necessary protection of the personnel of the pyrotechnic group, civilians, and the environment, not only from the blast wave, but also from the protective device itself (this causes the need for unloading holes in the case) and fragments (among other things, determines that the structure inside the protective device must prevent the flight of fragments through the outlets);
- the protective device has a mount for unloading operations when transported by a regular truck of the pyrotechnic division, if it has an appropriate manipulator;
- a protective device against a pyrotechnic group truck that brought a means of protection to the place of emergency, personal in protective equipment in the numbert of 2-3 people can deliver to the location of a dangerous object. Based on that, taking into account the requirement for labor protection for the maximum weight (up to 50 kg) that one pyrotechnician can carry, the weight should be about 100 kg, and this predetermines the need for handles in the case of a protective device for its transportation by personnel;
- taking into account that the layered structure provides the highest level of protection compared to other protective devices [11], the protective device has a domed shape. At the same time, the diameter of the protective device is less than 90 cm. This will ensure its passage through doorways [12] by pyrotechnicians, in the case when a dangerous object is inside a structure typical of a terrorist threat [13].

Based on that, the device should provide multiple uses in the localization of elements of destruction in the explosion of a small-sized dangerous object and for the transportation and destruction of explosive objects of different capacities by increasing the margin of safety, increasing mobility, simplifying the design, and reducing the cost of its manufacture and operation. That technical result is achieved due to the case being in the form of a dome with the possibility of placing explosive objects under it with handrails for ease of carrying and installation, and a structural element located around the perimeter of the base of the case for a tight fit to the surface. The case has a shape close to hemispherical and is formed by flat metal isosceles plates of trapezoidal and triangular shapes (Fig. 1).

The set of features provides the possibility of multiple safe uses of the localization device of the elements of the damage as a protective device for functional purposes by increasing its margin of strength. Simplification of the design reduces the cost of its manufacture and operation and simplifies the transportation process, which increases the mobility of the device.

Additional equipment for the determination of overpressure during field explosion tests on the localization device in accordance with Fig. 2 was installed. That made it possible to obtain indicators of the overpressure occurring inside the protective device. An MT-УХЛЗ type manometer was used.

It is important to note that the upcast (fixed by means of automatic photo and video capture) of the protective device additionally loaded in accordance with Table 2 did not exceed the height of passive embankment (load).

Comparison of the explosive test results in the coded variables, which are given in Table 3 (with the limit values), shows that the experimental results fall within the confidence interval. It was calculated with 0.95 reliability.

The following provisions must be taken into account:

The dome-shaped protective device is made of St 20 steel and has a diameter of 90 cm and a weight of 130 kg. For the local elimination of small explosive objects, it must have an additional load on the case;

To determine the additional load depending on the mass of a small-sized explosive object, it can use the results of the calculation in accordance with the mathematical model [8] to prevent emergencies associated with the threat of an explosion of a small-sized explosive object. The model uses a protective device with additional load (results in Table 2);

The dome-shaped protective device with a load to prevent the accidental departure of the fragments of an explosive object as a result of its detonation must be used with the passive embankment, which can be used as sandbags;

Twenty-five 50 kg sandbags must be available in the pyrotechnic division together with a dome-shaped protective device to prevent an emergency related to the risk of explosion of a small-sized explosive object (17 for a small-sized explosive object of 20 g TNT, including 8 for passive embankment).

### 3.2 Experimental method

A series of experimental fire tests on overpressure inside a prototype dome-shaped protective device (the device was made of St20 steel and was 90 cm in diameter with a mass of 130 kg) to verify the reliability of the mathematical model for preventing emergencies associated with the threat of the explosion small-sized object was carried out with an additional load as a result of the explosion of a small explosive object.

The experiment was based on the use of a protective device with an additional load on the case and further specification of the elimination features of explosive objects with its help. Experimental results for testing a mathematical model using standard statistical procedures have been obtained.

### 3.3 Сalculation method

It is expedient to use the results presented in coded form, taking into account the limited number of experimental results in the course of real explosion tests:

(1) $$x_{i}\left(X\right)=\frac{X_{calcul\;i}S-X_{actual\;i}}{X_{actual\;i}},$$

where ${Х}_{calcul\;i}$ – the *i*th design indicator of the protective device characteristic selected for verification. Based on the results of using the developed mathematical model for emergency warning, it was obtained with the initial data that correspond to the *i*th explosive test; ${Х}_{actual\;i}$– the actual value of the protective device selected for verification, obtained from the results of the *i*th explosion test.

This makes it possible to determine the reliability of the results obtained from the results of the developed mathematical model for preventing an emergency using the standard procedure [14] for checking whether the results of field experiments fall within confidence intervals calculated with reliability 0.95:

(2) $$x=\bar{x}\pm 1.96.\frac{\sigma_{x}}{\sqrt{n}},$$

where n– the number of protective device real tests to prevent emergencies associated with an unauthorized explosion of a small-size explosive object. Mathematical expectations $\bar{x}$ and standard deviations $\sigma_{x}$ from these results have been determined.

## Limitations

Not applicable.

## Ethics Statement

This research work does not involve studies with animals and humans.

## CRediT authorship contribution statement

**Denis Lyovin:** Formal analysis, Writing – original draft. **Victor Strelets:** Conceptualization, Software, Project administration, Writing – original draft. **Roman Shevchenko:** Methodology, Writing – review & editing. **Valentyna Loboichenko:** Writing – review & editing, Visualization. **Mikhail Divizinyuk:** Supervision. **Valery Strelets:** Resources, Investigation, Writing – original draft, Software. **Andrei Pruskyi:** Validation, Data curation.

## Data Availability

Experimental_results for dome-shaped protective device (Original data) (Mendeley Data)
